# Optimizing water additives during live transportation of Pangasius catfish (*Pangasianodon hypophthalmus*): Effects on water quality, bacterial regrowth, and stress response

**DOI:** 10.5455/javar.2026.m1049

**Published:** 2026-06-22

**Authors:** Md. Nurul Haider, Md. Nazmul Islam Rifat, Maliha Afsana, Abdul Kader Jilani, Md. Adil Mahfuz, Md. Mubarack Hossain

**Affiliations:** 1 Department of Fisheries Technology, Faculty of Fisheries, Bangladesh Agricultural University, Mymensingh-2202, Bangladesh

**Keywords:** Water additives, bacterial regrowth, water quality parameters, live transportation, Pangasius catfish

## Abstract

**Background:** In Bangladesh, Pangasius catfish (*Pangasianodon hypophthalmus*) is commonly transported live from production sites to retail markets. Poor water quality and bacterial regrowth during transportation pose major challenges for animal welfare and fish quality.

**Objectives:** This study evaluated the effects of some water additives such as salt (NaCl), Electromin Saline (ES), and methylene blue (MB) on water quality parameters, bacterial regrowth, and stress responses of Pangasius catfish during live transportation.

**Materials and Methods:** Two experimental trials were conducted: an on-farm trial under controlled conditions and a field trial during commercial transport. In both the trials, tanks containing live fish were treated with additives individually and in combination, while untreated tanks served as controls. Water temperature, pH, dissolved oxygen, ammonia, viable bacterial counts, and blood glucose were measured.

**Results:** Treated tanks showed improved water quality, reduced ammonia accumulation, and significantly lower bacterial regrowth compared with controls. Blood glucose levels were also reduced in treated fish, indicating reduced physiological stress during transportation. Among the treatments, 2.0 ppt salt, 2.0 ppt ES, and their combinations with MB (1.0 ppm) effectively improved water quality, suppressed bacterial regrowth, and reduced transportation-induced stress during live transportation of Pangasius catfish.

**Conclusions:** Salt and ES at 2.0 ppt, alone or combined with methylene blue, can effectively be used during live fish transportation.

## 1. Introduction

Fish transportation in live conditions after harvesting at some point before retailing is a global trend associated with human nutrition [1]. Since live fish generally command higher market prices and enjoy strong consumer preference, efficient transport from production sites to regional markets has become an essential component of the supply chain. In Bangladesh, Pangasius catfish (*Pangasianodon hypophthalmus*) has become an important fish food due to the volumes produced and for its accessibility to lower-income consumers. This species is commonly transported alive from farms to retail markets across the country to satisfy the prevailing preference for fresh, live fish [2]. The stress associated with live transportation is extensively documented, with a strong focus on maintaining water quality to reduce it [3]. Multiple stressors during transport, including overcrowding, turbulence, and deteriorating water quality, can cause mucus loss, tissue damage, reduced immunity, and, in severe cases, mortality [4]. In Bangladesh, inefficient handling and suboptimal transport methods have been linked to significant mortality during shipment [5], underscoring the need for well-designed transport protocols to protect fish health and economic returns [6].

Recent studies have highlighted that live fish transportation remains a critical bottleneck in aquaculture supply chains, particularly in developing countries where infrastructure and handling practices are often suboptimal. Transport-induced stress results from multiple interacting factors, including crowding, hypoxia, ammonia accumulation, and microbial proliferation, all of which compromise fish welfare and post-transport survival [3, 7]. Prolonged overcrowding, agitation, and physical injuries during transport can further exacerbate stress, increase susceptibility to bacterial infections, and raise mortality risk [8]. It is widely reported that substantial numbers of fish die during transport due to stress before reaching the market [9]. During transport, metabolic activity and respiration lead to CO_2_ accumulation in the water. High CO_2_ concentrations impair oxygen uptake and, upon dissolving in water, form carbonic acid, which lowers plasma pH and disrupts normal physiological processes [10]. This effect is compounded by the accumulation of toxic ammonia (NH_3_), which can severely limit loading density and survival [9]. Stress responses in fish occur in three stages: primary (cortisol release), secondary (elevated blood glucose, a common stress indicator), and tertiary (long-term physiological adjustments if stress persists) [6, 11, 12, 13, 14].

Deterioration in water quality not only stresses fish but also increases their vulnerability to bacterial infections by damaging mucus layers and scales [15]. Bacteria may enter the transport system via water and fish, with density and diversity increasing along the transport route [9, 16]. Some bacterial species act as opportunistic pathogens, causing spoilage and lowering the market value of live fish.

In recent years, researchers have increasingly focused on using water additives to mitigate these stressors. Water quality during live transport can be improved by using additives such as sodium chloride (NaCl) and methylene blue (MB, C_16_H_18_N_3_ClS·3H_2_O) [17]. Other additives include glucose, antibiotics, and probiotics, which are used to reduce stress and injury during transport, though their effectiveness varies by species and conditions [18, 19, 20]. Sodium chloride (NaCl) is widely reported to improve osmoregulatory balance and reduce physiological stress in freshwater fish during transport [20, 21]. NaCl is inexpensive, readily available, safe for handlers, and approved for use in aquaculture, including transport [17, 20]. In freshwater fish, NaCl helps maintain electrolyte balance (Na^+^ and Cl^-^ ions), reduces osmotic stress, enhances welfare, and lowers disease incidence [22, 23].

Similarly, antimicrobial agents such as methylene blue (MB) have been shown to suppress microbial growth and reduce oxygen demand in transport water [24]. According to the National Library of Medicine, methylene blue functions as an oxidation–reduction agent capable of inhibiting bacterial and microalgal regrowth. Studies indicate that NaCl and MB together are more effective at reducing bacterial load than when used separately [17, 24].

Furthermore, emerging studies suggest that combined additive approaches may provide synergistic benefits by simultaneously improving water quality and inhibiting bacterial proliferation. For example, Hong et al. [25] demonstrated that water quality deterioration is closely linked to stocking density and microbial activity, while Urakawa and Sipos [19] highlighted the importance of microbial management in transport systems. Despite these advances, limited studies have evaluated the combined effects of electrolyte-based additives and antimicrobial agents under both controlled and commercial transport conditions.

Although several studies have examined the use of individual additives during fish transport, limited information is available on their combined effects under both controlled and commercial transport conditions. Therefore, this study addresses this gap by systematically evaluating the individual and combined effects of salt, commercial saline, and methylene blue on water quality, bacterial regrowth, and physiological stress responses during live transportation of Pangasius catfish under both on-farm and field conditions. The present study aimed to evaluate the effectiveness of selected water additives in improving water quality, reducing bacterial regrowth, and mitigating stress during live transportation of Pangasius catfish.

## 2. Materials and Methods

### 2.1. Animal welfare and ethical approval

The study protocol was approved, and the research was performed according to the guidelines of the Animal Welfare and Experimentation Ethics Committee (AWEEC) of Bangladesh Agriculture University in Mymensingh [AWEEC/BAU/2024 (32)].

### 2.2. Study sites

This experiment was conducted in two phases. The first phase, referred to as the on-farm experiment, was carried out at Fulbaria Upazila, Mymensingh, Bangladesh. The second phase, termed the field trial, was conducted along a commercial supply route from Trishal, Mymensingh, to a fish market in Dhaka, located approximately 100 km away, with a transportation time of about 6 h. Laboratory analyses were performed in the Fisheries Microbiology Laboratory, Department of Fisheries Technology, Faculty of Fisheries, Bangladesh Agricultural University, Mymensingh.

### 2.3. Fish harvesting and experimental setup

**Figure 1.**
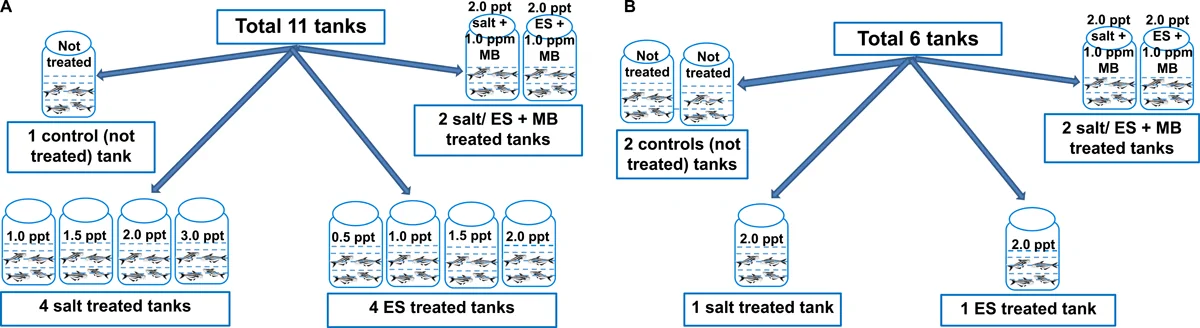
Experimental setup with live Pangasius catfish (*P. hypophthalmus*) A. on-farm in laboratory conditions and B. during live transportation in a supply channel from Mymensingh to Dhaka, Bangladesh.

On-farm experiment: Pangasius catfish were harvested using a surrounding net and acclimatized within a net enclosure. Eleven plastic tanks (4 ft length × 1 ft width) were placed under shade at the farm, each loaded with 4 kg of Pangasius catfish (4 fish) in 20 L of deep tube-well water. Of the 11 tanks, four were treated with salt at final concentrations of 1.0, 1.5, 2.0, and 3.0 ppt; four with commercially available saline (Electromin powder, Square Pharmaceuticals Ltd., Bangladesh; containing sodium bicarbonate, sodium chloride, potassium chloride, dextrose, vitamin A, zinc sulfate, calcium chloride, and manganese sulfate) at final concentrations of 0.5, 1.0, 1.5, and 2.0 ppt; and two with a combination of salt or ES plus MB (2.0 ppt salt + 1.0 ppm MB and 2.0 ppt ES + 1.0 ppm MB). One tank served as an untreated control (Figure 1A). The treatments were determined based on previous studies [24] and common practices during live fish transportation at the field level. During transport, no artificial aeration or water exchange was applied, and tanks were maintained under ambient environmental conditions to simulate typical commercial transport practices. The tanks were monitored for 8 h, and water sub-samples were collected at 2 h intervals for physicochemical analysis and bacterial counts.

Field trial: Conducted during commercial live transport of Pangasius catfish from Mymensingh to Dhaka. Six fish-holding tanks, each containing 40 kg of fish (about 25–26 individuals, ~1.5 kg each) with 180 L of water, were loaded onto a transport truck. The most effective additive doses identified in the on-farm experiment were applied: 2.0 ppt salt in one tank, 2.0 ppt ES in one tank, and a combination of 2.0 ppt salt + 1.0 ppm MB or 2.0 ppt ES + 1.0 ppm MB in two tanks. Two tanks without additives served as control and control 2, respectively (Figure 1B). The treatments selected for the field trial were based on their performance in the on-farm experiment, considering their effectiveness in maintaining higher dissolved oxygen, reducing ammonia accumulation, lowering bacterial counts, and minimizing blood glucose levels. Two control tanks were included to represent independent controls under field conditions and to account for variability associated with commercial transportation. Water samples were collected at 2 h intervals until fish were unloaded at the retail market. Sterile 250 ml plastic bottles were used for sampling; physicochemical measurements were taken immediately, and bacterial counts were initiated on the truck.

Each tank was considered the experimental unit. Due to logistical constraints associated with handling large fish and simulating commercial transport conditions, treatments were conducted using a single tank per treatment. However, repeated measurements were taken at multiple time points (0, 2, 4, 6, and 8 h), and these temporal observations were incorporated into the statistical analysis to evaluate treatment effects over time.

### 2.4. Determination of physicochemical parameters

The physicochemical parameters such as water temperature, DO, pH, and NH_3_ were measured as described in Hossain et al. [16]. Water temperature was determined by a mercury glass rod thermometer. The DO content of the water was determined using a DO test kit (Biosol, AA Biotech, India) according to the manufacturer’s protocol. Briefly, the test bottle was rinsed with sample water, filled to overflow, and treated sequentially with DO-1 and DO-2 reagents. After settling, DO-3 was added to dissolve precipitates, followed by DO-4. Finally, DO-5 was added dropwise until the blue color disappeared, and DO content was calculated (Equation 1).

The pH of the water was determined by using a pH test kit (CP-102, Thailand). Five milliliters of sample water were placed in a clean color cell, two drops of pH reagent were added, and the resulting color was compared to a standard chart.

Total ammonia content was determined using a total ammonia test kit (AQUA AM). Five milliliters of sample water were treated sequentially with Ammonia-1, Ammonia-2, Ammonia-3, and Ammonia-4 reagents. After 20 min, color intensity was compared to the kit’s reference chart to determine ammonia concentration.

Commercial test kits were used due to their practicality, rapid application, and widespread use in aquaculture field conditions. All measurements were conducted following the manufacturers’ instructions. Although these kits may be less precise than laboratory-based instrumental methods, they are suitable for comparative assessments under field conditions and already used in scientific studies [2].

### 2.5. Estimation of the viable bacterial counts

Total viable bacterial counts were determined using the drop plate method on Plate Count Agar (HiMedia, India). Water samples were serially diluted in sterile physiological saline (0.85% NaCl), and 10 µl of each dilution was inoculated onto agar plates in duplicate. Plates were incubated at 30 °C for 24 h. Colonies ranging from 3 to 30 per drop were counted, and results were expressed as colony-forming units (CFU/ml), representing total viable bacteria.

For this purpose, 100 µl of collected water sub-samples were mixed with 900 µl of previously prepared physiological saline in an Eppendorf tube. Then, 10 µl of each tenfold diluted water sample was transferred and dropped (the drop plate method was used) onto agar plates maintaining duplicates, using a micropipette for each dilution. Agar plates were prepared previously with Plate Count agar media (HiMedia, India). The cultured agar plates were then kept in an inverted position in a zipper bag and transferred to the laboratory and incubated at 30 °C for 24 h. After incubation, plates with 3 to 30 colonies in each segment of the agar plates were considered. The average number of colonies in a particular dilution was multiplied with a dilution factor to obtain the viable count of bacteria. The result of the viable count of bacteria was expressed as the colony-forming unit (CFU) per ml of the water sample and calculated using Equation 2.

### 2.6. Determination of the blood glucose

Blood glucose was determined at 0 h (just after loading the fishes) and at the end of the experiment period (after 8 h/before unloading the fishes in the retail market) by using a glucometer (Bioland, China). Blood was collected by pricking along the lateral line with a sterile lancet, and the droplet was applied to a glucometer test strip. Readings were displayed within seconds. Blood glucose was used as a secondary stress indicator, as it is widely recognized as a rapid and reliable measure of physiological stress in fish during handling and transport.

### 2.7. Statistical analysis

Data were recorded and analyzed using Microsoft Excel 2013 and SPSS (version 26). A two-way analysis of variance (ANOVA) was performed to evaluate the effects of treatment, time, and their interaction on the measured parameters. Where significant differences were detected, Tukey’s HSD post-hoc test was applied for multiple comparisons among treatment means. Differences were considered statistically significant at *p* < 0.05.

Due to logistical constraints, each treatment was represented by a single tank; therefore, the tank was considered the experimental unit, and repeated measurements over time were treated as temporal observations rather than independent biological replicates. Consequently, statistical results should be interpreted with caution, particularly regarding treatment effects.

### 2.8. Equations

1. DO (ppm) = 0.65 × (No. of drops of DO 5)
2. Viable count of bacteria in water (CFU/ml) = average no. of colonies on agar plate from each drop × dilution factor × 100

## 3. Results

### 3.1. Changes in physicochemical parameters

Especially in the case of physicochemical parameters, relatively large F-values observed for certain parameters (e.g., dissolved oxygen and ammonia) reflect strong temporal trends combined with low within-treatment variability; however, given the limited biological replication, these results should be interpreted as indicative rather than definitive.

#### 3.1.1. Changes in temperature

**Figure 2.**
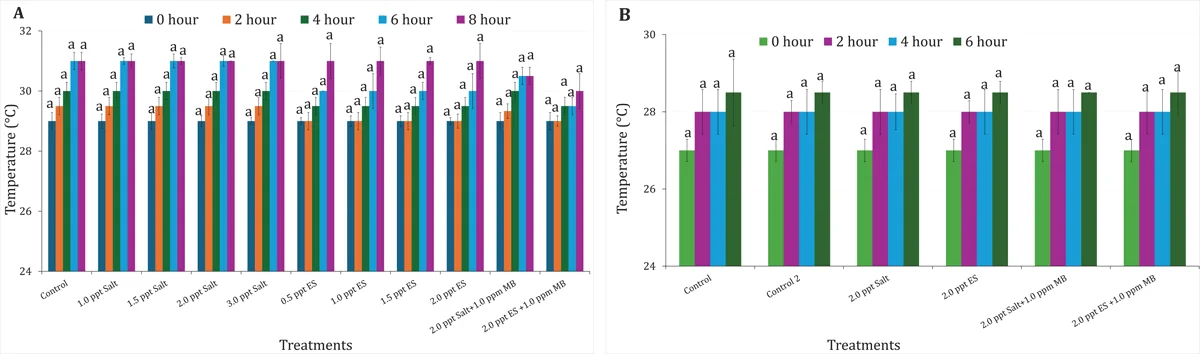
Fluctuations in temperatures of the water within the experimental tanks at different time intervals: A. in the case of an on-farm experiment and B. a field trial during live transportation of Pangasius catfish (*P. hypophthalmus*) treated with different water additives. Error bars indicating standard errors (SEs) and different superscript letters (e.g., ^a, b, c,^ etc.) within time points refer to significant differences between them at the 5% level (*p* < 0.05), as determined by two-way ANOVA with post-hoc Tukey comparisons.

Water temperature showed only minor fluctuations (29.0 ± 0.289 °C to 31.0 ± 0.289 °C in the on-farm experiment and from 27.0 ± 0.289 °C to 28.5 ± 0.866 °C in the field trial) over time in both experiments and remained within a narrow range across treatments, with no consistent treatment-specific effects observed (Figure 2).

A two-way ANOVA was conducted to test the effects of treatment (control, 1.0 ppt salt, 1.5 ppt salt, 2.0 ppt salt, 3.0 ppt salt, 0.5 ppt ES, 1.0 ppt ES, 1.5 ppt ES, 2.0 ppt ES, 2.0 ppt salt + 1.0 ppm MB, and 2.0 ppt ES + 1.0 ppm MB in the case of the on-farm experiment; and control, control 2, 2.0 ppt salt, 2.0 ppt ES, 2.0 ppt salt + 1.0 ppm MB, and 2.0 ppt ES + 1.0 ppm MB in the case of the field trial) and time (0, 2, 4, 6, and 8 h in the case of the on-farm experiment; and 0, 2, 4, and 6 h in the case of the field trial) on physicochemical parameters of transport water. In the case of the on-farm experiment, finding showed main effects of time (F = 86, *p* < 0.001) and treatment (F = 3.01, *p* = 0.003), but no interaction (treatment × time: F = 0.80, *p* = 0.775). Tukey HSD within each sampling hour indicated small but detectable differences across treatments at later time points (Figure 2A).

On the other hand, in the case of field trial effects, time was significant (F = 11, *p* < 0.001). However, there were no effects of treatment (F = 0.49, *p* = 0.779) or the interaction (treatment × time: F = 1.21, *p* = 0.288). Tukey showed usual temporal warming with no consistent treatment separation at each time (Figure 2B).

#### 3.1.2. Changes in DO

**Figure 3.**
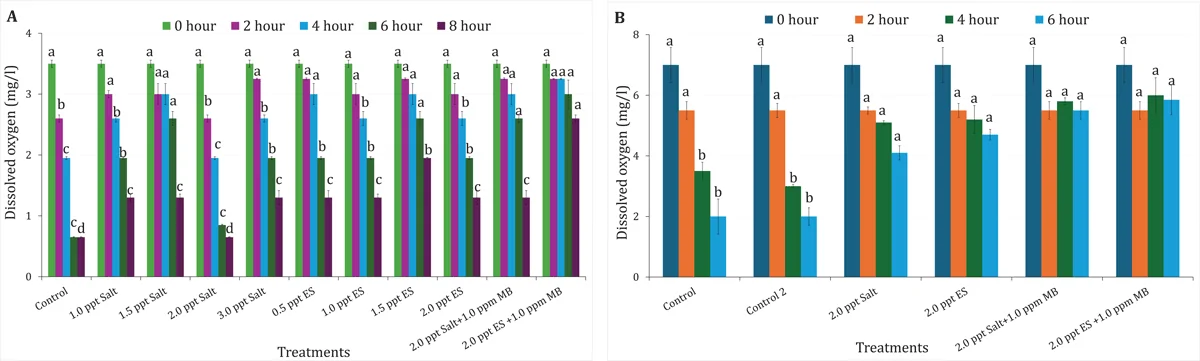
Changes in dissolved oxygen (DO) levels of water in the experimental tanks at different time intervals: A. in the case of the on-farm experiment and B. field trial during live transportation of Pangasius catfish (*P. hypophthalmus*) treated with different water additives. Error bars indicating standard errors (SEs) and different superscript letters (e.g., ^a, b, c,^ etc.) within time points refer to significant differences between them at the 5% level (*p* < 0.05), as determined by two-way ANOVA with post-hoc Tukey comparisons.

Dissolved oxygen (DO) declined progressively over time in all treatments, with a more pronounced reduction in the untreated controls (Figure 3). Treatments containing additives, particularly combinations with methylene blue, consistently maintained higher DO levels compared with single-additive and control groups. The effect became more evident at later sampling points, indicating a time-dependent treatment influence.

Outputs of two-way ANOVA with post hoc showed significant effects of time (F = 2455.17, *p* < 0.001), treatment (F = 187, *p* < 0.001), and their interaction (F = 13, *p* < 0.001) in the case of the on-farm experiment. At 8 h, the highest DO was in 2.0 ppt ES + 1.0 ppm MB (2.60 ± 0.06 mg/l; letter “a”), with 1.5 ppt ES (1.95 ± 0.01 mg/l; “b”) next; the untreated control was among the lowest (≈0.65 ± 0.01 mg/l; far-separated letter) (Figure 3A). A similar trend was obtained in the case of an on-field trial. All time (F = 1,061, *p <* 0.001), treatment (F = 87, *p* < 0.001), and their interaction (treatment × time: F = 4.69, *p* < 0.001) had significant effects. At 6 h, the highest value of DO was found in 2.0 ppt ES + 1.0 ppm MB-treated tanks (5.85 ± 0.49 mg/l; “a”), far higher than controls (~2.0 ± 0.3–0.6 mg/l) (Figure 3B).

#### 3.1.3. Changes in pH

**Figure 4.**
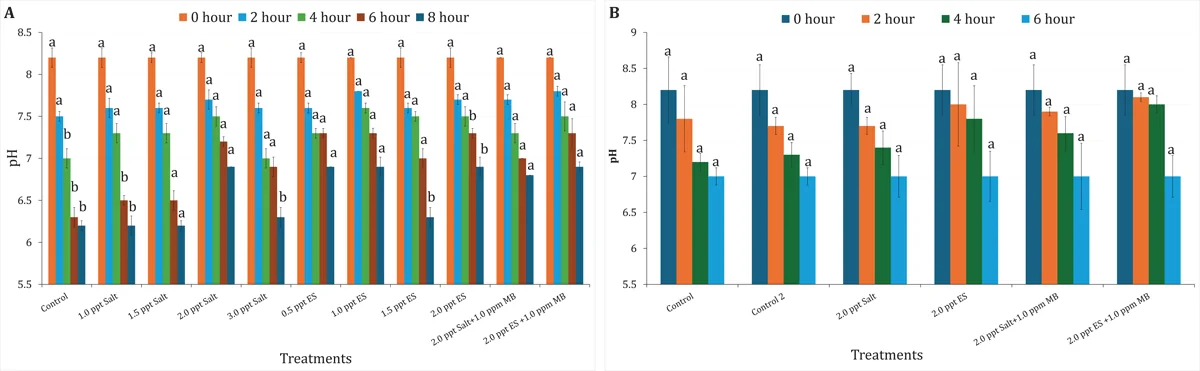
Changes in pH values of water in the experimental tanks at different time intervals: A. in the case of the on-farm experiment and B. field trial during live transportation of Pangasius catfish (*P. hypophthalmus*) treated with different water additives. Error bars indicating standard errors (SEs) and different superscript letters (e.g., ^a, b, c,^ etc.) within time points refer to significant differences between them at the 5% level (*p* < 0.05), as determined by two-way ANOVA with post-hoc Tukey comparisons.

Initial pH values were relatively high and similar across treatments but decreased steadily over time. Slightly lower pH was recorded in the untreated controls. By the end, pH dropped from 8.2 ± 0.115 to 6.2 ± 0.058 after 8 h in the on-farm experiment and from 8.20 ± 0.46 to approximately 7.00 ± 0.12 after 6 h in the field trial (Figure 4).

Two-way ANOVA showed there were significant effects of time (F = 318, *p* < 0.001), treatment (F = 3.59, *p* < 0.001), and their interaction (treatment × time: F = 6.48, *p* < 0.001) in the case of the on-farm experiment. By 8 h, Tukey grouping showed higher pH in additive treatments than control; combinations with MB generally maintained higher pH than their single-additive counterparts (Figure 4A).

In case of a field trial, on the other hand, there was no significant main effect of treatment (F = 0.57, *p* = 0.7189) or treatment × time interaction (F = 0.24, *p* = 0.9977). However, time had a significant main effect (F = 17, *p* = < 0.001), indicating that pH declined during the transport period. Post-hoc Tukey comparisons of treatments show no significant treatment differences within time points (Figure 4B).

#### 3.1.4. Changes in ammonia

**Figure 5.**
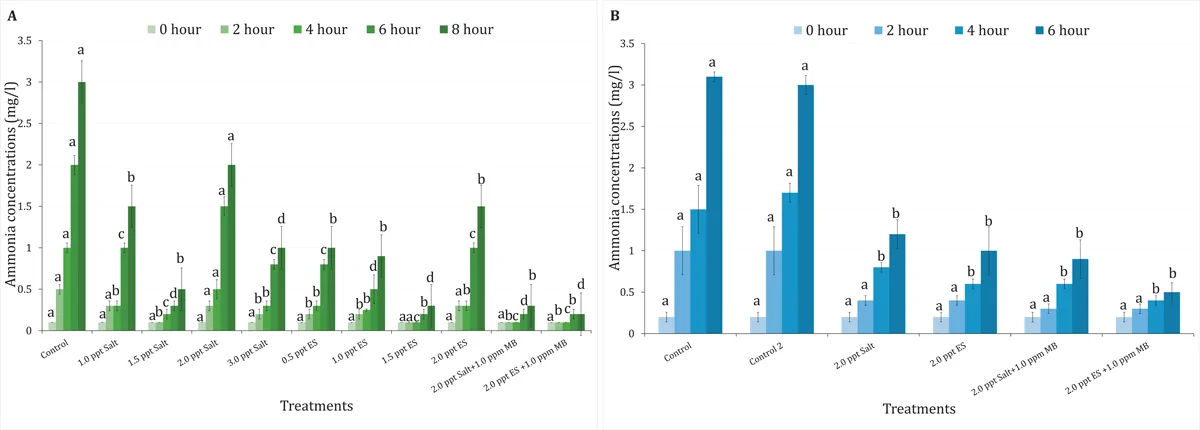
Fluctuations in ammonia concentrations of water in the experimental tanks at different time intervals: A. in the case of the on-farm experiment and B. in the field trial experiment during live transportation of Pangasius catfish (*P. hypophthalmus*) treated with different water additives. Error bars indicating standard errors (SEs) and different superscript letters (e.g., ^a, b, c,^ etc.) within time points refer to significant differences between them at the 5% level (*p* < 0.05), as determined by two-way ANOVA with post-hoc Tukey comparisons.

Ammonia concentrations increased steadily over time in all treatments, with the highest accumulation observed in control tanks (Figure 5). Additive treatments, particularly combinations of salt or saline with methylene blue, effectively suppressed ammonia buildup, with differences becoming more distinct at later stages of transport.

In the case of the on-farm experiment, strong main effects of time (F = 2259, *p* < 0.001), treatment (F = 500, *p* < 0.001), and their interaction (treatment × time: F = 41, *p* < 0.001) were observed. At 8 h, control had the highest ammonia (≈3.0–4.0 mg/l), whereas 2.0 ppt ES + 1.0 ppm MB and 2.0 ppt salt + 1.0 ppm MB were lowest (≈0.20–0.30 mg/l), indicating a significant increase in ammonia over single-additive and control tanks (Figure 5A).

In the case of the field trial, similar significant effects were observed for time (F = 2506, *p* < 0.001), treatment (F = 373, *p* < 0.001), and treatment × time interaction (F = 51, *p* < 0.001). At 6 h, ammonia was lowest in 2.0 ppt ES + 1.0 ppm MB (0.50 ± 0.12) and 2.0 ppt salt + 1.0 ppm MB (0.90 ± 0.23), intermediate in 2.0 ppt ES/salt (≈1.0–1.2 mg/l), and highest in controls (≈3.0–3.1 mg/l), with non-overlapping Tukey letters (Figure 5B).

### 3.2. Changes in the viable count of bacteria

Viable bacterial counts increased in control tanks over time, whereas treated groups showed stable or reduced counts (Tables 1, 2). The greatest reductions were observed in treatments combining methylene blue with salt or saline, indicating enhanced antimicrobial effectiveness compared with single-additive treatments.

**Table 1.** Changes in viable bacterial counts (means ± SEs) as colony-forming units (CFUs)/ml of water in the case of an on-farm experiment. The water tanks with live Pangasius catfish (*P. hypophthalmus*) were treated with different water additives and observed for a period of 8 h.

Time	Untreated/Control (Mean ± SE)	Viable bacterial counts (× 10^4^) CFU/ml											
		Salt treated					ES treated			Combination with Methylene Blue	
		1.0 ppt (Mean ± SE)	1.5 ppt (Mean ± SE)	2.0 ppt (Mean ± SE)	3.0 ppt (Mean ± SE)	0.5 ppt (Mean ± SE)	1.0 ppt (Mean ± SE)	1.5 ppt (Mean ± SE)	2.0 ppt (Mean ± SE)	2.0 ppt salt + 1.0 ppm MB (Mean ± SE)	2.0 ppt ES + 1.0 ppm MB (Mean ± SE)
0 h	2.20 ± 0.14^ab^	2.40 ± 0.28^c^	2.60 ± 0.42^b^	2.05 ± 0.21^b^	2.35 ± 0.21^ab^	2.30 ± 0.70^a^	2.40 ± 0.42^b^	2.00 ± 0.56^a^	2.15 ± 0.63^a^	2.70 ± 0.56^b^	2.10 ± 0.70^a^
2 h	2.40 ± 0.14^bc^	2.20 ± 0.14^bc^	2.00 ± 0.28^ab^	1.70 ± 0.28^b^	2.00 ± 0.14^a^	1.75 ± 0.21^a^	2.00 ± 0.28^b^	1.90 ± .014^a^	1.65 ± 0.35^a^	1.90 ± 0.28^ab^	1.45 ± 0.21^a^
4 h	2.70 ± 0.14^bc^	1.8 ± 0.14^abc^	2.05 ± 0.21^ab^	1.65 ± 0.21^ab^	2.40 ± 0.28^ab^	1.70 ± 0.14^a^	1.90 ± 0.14^ab^	1.70 ± 0.28^a^	1.40 ± 0.28^a^	1.40 ± 0.42^ab^	1.35 ± 0.35^a^
6 h	1.45 ± 0.35^a^	1.45 ± 0.21^ab^	1.40 ± 0.42^ab^	1.20 ± 0.14^ab^	2.80 ± 0.14^b^	1.50 ± 0.14^a^	1.45 ± 0.00^ab^	1.30 ± 0.07^a^	1.25 ± 0.21^a^	1.20 ± 0.42^ab^	1.05 ± 0.35^a^
8 h	3.10 ± 0.14c	1.25 ± 0.21^a^	1.15 ± 0.21^a^	0.95 ± 0.21^a^	3.00 ± 0.14^b^	1.20 ± 0.14^a^	1.00 ± 0.14^a^	0.85 ± 0.50^a^	0.70 ± 0.28^a^	0.90 ± 0.28^a^	0.75 ± 0.21^a^
*p*-value	0.003***	0.010***	0.035*	0.021*	0.019*	0.153	0.014*	0.066	0.086	0.039*	0.127

***Refers it being significant at 1% level. *Refers to its significance at the 5% level. Different superscript letters (e.g., ^a, b, c,^ etc.) refer to significant differences between them.

**Table 2.** Changes in viable bacterial counts (means ± SEs) as colony-forming units (CFUs)/ml of water in the case of the field trial. The water tanks with live Pangasius catfish (*P. hypophthalmus*) were treated with different water additives and observed for a period of 6 h.

Time	Viable bacterial counts (× 10^4^) CFU/ml					
	Untreated/Control (Mean ± SE)		Salt treated (Mean ± SE)	ES treated (Mean ± SE)	Combination with Methylene Blue (Mean ± SE)	
	Control	Control 2	2.0 ppt salt	2.0 ppt ES	2.0 ppt salt + 1.0 ppm MB	2.0 ppt ES + 1.0 ppm MB
0 h	2.33 ± 0.17	2.30 ± 0.25	2.28 ± 0.17	2.30 ± 0.18	2.23 ± 0.22	2.20 ± 0.21
2 h	2.48 ± 0.22	2.45 ± 0.23	2.18 ± 0.22	2.13 ± 0.25	2.08 ± 0.22	1.95 ± 0.12
4 h	2.63 ± 0.35	2.65 ± 0.23	2.08 ± 0.17	2.00 ± 0.29	1.85 ± 0.12	1.53 ± 0.17
6 h	3.00 ± 0.18	2.98 ± 0.22	1.68 ± 0.12	1.45 ± 0.47	1.15 ± 0.26	0.83 ± 0.17
*p*-value	0.01*					

* It refers to its significance at the 5% level.

A gradual increase of bacterial loads in the control/untreated tank of the on-farm experiment was observed; the initial viable count (mean ± SE) was 2.2 ± 0.14 × 10^4^ CFU/ml, which became 3.1 ± 0.14 × 10^4^ CFU/ml at the end (*p* = 0.003) of the experiment (at 8 h). In most treated tanks, counts significantly decreased from the initial values (Table 1). The combined use of salt/ES and methylene blue-treated tanks was found efficient in lowering the bacterial viable counts compared to others, which were 2.7 ± 0.56 × 10^4^ CFU/ml to 0.9 ± 0.28 × 10^4^ CFU/ml in 2.0 ppt salt + 1.0 ppm MB-treated and 2.1 ± 0.70 × 10^4^ CFU/ml to 0.75 ± 0.21 × 10^4^ CFU/ml in 2.0 ppt ES + 1.0 ppm MB-treated tanks (Table 1).

In the field trial, similar trends were observed. Initial bacterial counts were about 2.33 ± 0.17 × 10^4^ CFU/ml in all tanks. Counts increased in untreated tanks to 3.0 ± 0.18 × 10^4^ CFU/ml (control) and 2.98 ± 0.22 × 10^4^ CFU/ml (control 2) after 6 h (Table 2). In treated tanks, viable counts decreased significantly (*p* = 0.01). Tanks treated with a combination of MB showed better reductions than those treated with only salt/ES, with decreases from 2.23 ± 0.22 × 10^4^ CFU/ml to 1.15 ± 0.26 × 10^4^ CFU/ml (2.0 ppt salt + 1.0 ppm MB) and from 2.2 ± 0.21 × 10^4^ CFU/ml to about 0.83 ± 0.17 × 10^4^ CFU/ml (2.0 ppt ES + 1.0 ppm MB) (Table 2).

### 3.3. Observation of the blood glucose levels of the experimental fishes

Blood glucose levels increased in all treatments during transport, with significantly higher values in control groups (Table 3). Fish exposed to additive treatments, particularly those containing methylene blue, exhibited comparatively lower glucose levels, indicating reduced physiological stress.

**Table 3.** Changes of blood glucose levels (mmol/l) in Pangasius catfish (*P. hypophthalmus*) in the case of on-farm and field trials. The test was conducted just before loading (at 0 h) and at the end of the experiment period (after 8 h of observation in the case of on-farm and after 6 h in the case of the field trial).

Experimental periods	Treatment	Blood glucose levels* (mmol/l) On-farm experiment (Mean ± SE)	Blood glucose levels (mmol/l) On-field experiment (Mean ± SE)
At 0 h (initial before treatment)		1.70 ± 0.45^f^**	4.98 ± 0.20^d^
After 6–8 h	Control	7.27 ± 0.15^a^	7.08 ± 0.11^a^
	Control 2	Not applicable	7.17 ± 0.19^a^
	1.0 ppt salt	6.37 ± 0.07^b^	Not applicable
	1.5 ppt salt	5.94 ± 0.25^c^	Not applicable
	2.0 ppt salt	5.10 ± 0.11^d^	6.58 ± 0.11^a^
	3.0 ppt salt	6.63 ± 0.09^b^	Not applicable
	0.5 ppt ES	5.66 ± 0.05^c^	Not applicable
	1.0 ppt ES	5.42 ± 0.03^c^	Not applicable
	1.5 ppt ES	5.27 ± 0.02^d^	Not applicable
	2.0 ppt ES	5.15 ± 0.08^d^	6.47 ± 0.15^b^
	2.0 ppt salt + 1.0 ppm MB	5.17 ± 0.09^d^	6.12 ± 0.08^b^
	2.0 ppt ES + 1.0 ppm MB	4.53 ± 0.18^e^	5.70 ± 0.15^c^
	*p*-value	0.000***	0.000***

*Values are presented as mean ± standard error (SE). ***Refers to statistically significant at the 1% level. Different superscript letters (e.g., ^a, b, c,^ etc.) in the same column refer to significant differences between them at the 5% level (*p* < 0.05), as determined by two-way ANOVA with post-hoc Tukey comparisons.

In the on-farm experiment, treatment had a significant effect on blood glucose after 8 h (F = 46, *p* < 0.001). The highest mean (± SE) occurred in control (7.27 ± 0.15^a^), followed by 3.0 ppt salt (6.63 ± 0.09^b^) and 1.0 ppt salt (6.37 ± 0.07^b^). Intermediate and lower values were observed for 1.5 ppt salt (5.94 ± 0.25^c^), 0.5 ppt ES (5.66 ± 0.05^c^), 1.0 ppt ES (5.42 ± 0.03^c^), 1.5 ppt ES (5.27 ± 0.02^d^), 2.0 ppt salt + 1.0 ml MB (5.17 ± 0.09^d^), 2.0 ppt ES (5.15 ± 0.08^d^), 2 ppt salt (5.10 ± 0.11^d^), and the lowest in 2.0 ppt ES + 1.0 ml MB (4.53 ± 0.18^e^). Notably, the ES + MB combination (2.0 ppt ES + 1.0 ml MB) produced significantly lower glucose than control and all salt-only treatments (Tukey-adjusted *p* < 0.05), indicating reduced stress (Table 3).

In the field trial, treatment also significantly affected blood glucose after 6 h (F = 17, *p* < 0.001). Mean (± SE) values were control 2 (7.17 ± 0.19^a^) ≈ control (7.08 ± 0.11^a^) ≈ 2.0 ppt salt (6.58 ± 0.11^a^), 2.0 ppt ES (6.47 ± 0.15^b^), 2.0 ppt salt + 1.0 ml MB (6.12 ± 0.08^b^), and the lowest 2.0 ppt ES + 1.0 ml MB (5.70 ± 0.15^c^). In this case also, the ES + MB treatment (2.0 ppt ES + 1.0 ml MB) was significantly lower than both controls (adjusted *p* < .001), again consistent with reduced transport stress (Table 3).

## 4. Discussion

Live fish transportation is a common practice due to consumer preference for fresh fish and the higher market price it commands. However, minimizing transport-related stress in fish and reducing mortality remains a challenge. When a large number of fish are packed together, water quality deteriorates rapidly—dissolved oxygen drops, metabolic wastes and bacterial concentrations increase, stress responses are triggered, and overall fish quality declines. The present study evaluated the influence of salt, ES, and methylene blue (MB), alone and in combination, on water quality and physiological responses of *Pangasius hypophthalmus* during live transport, both under on-farm and field conditions. Across both experiments, highly significant effects of treatment, time, and treatment × time interactions were observed for most parameters, underscoring that additive benefits became more pronounced with increasing transport duration.

During the study period, water temperature ranged from 27 °C to 31 °C, which is within the favorable limits for Pangasius catfish and was not significantly affected by the additives (Figure 2). This range also provides favorable conditions for bacterial growth, as higher temperatures accelerate metabolic activities and promote bacterial proliferation [9]. Among other water quality parameters, DO levels fluctuated, sometimes reaching critical levels in the untreated tanks (Figure 3). DO levels declined progressively over time in all treatments, reflecting continuous oxygen consumption through fish respiration and microbial activity. However, the higher DO levels observed in MB-treated groups may be associated with reduced microbial activity, as suggested by previous studies [25, 26], although this mechanism was not directly measured in the present study.

The significant treatment × time interaction in both on-farm and field trials highlights that additive effects were minimal at 0 h but became increasingly evident by 4–6 h, when DO in control tanks had dropped sharply.

Water pH is another important and sensitive factor, influenced mainly by CO_2_ and ammonia accumulation during transport. Gomes et al. [6] reported that a pH below 6.0–6.5 can be detrimental to fish during transport without water exchange. In our study, transport stress caused a gradual decline in pH, primarily due to CO_2_ accumulation and organic acid production. While all treatments showed pH reduction with time, fish in the MB + salt or MB + ES groups retained a relatively higher and more stable pH. This suggests that MB’s antimicrobial properties mitigated bacterial fermentation, reducing acidification. Notably, differences were stronger in the on-farm trial than in the field trial, where environmental variability (e.g., fluctuating ambient temperature, variable aeration) may have dampened treatment effects (Figure 4). The decline in pH is consistent with findings by Wurts [27] and Singh et al. [28], who reported that CO_2_ reacts with water to form carbonic acid, releasing hydrogen ions and lowering pH. Although this pH range was safe for fish survival, it is still optimal for bacterial growth, highlighting the need for antibacterial measures during transport.

Ammonia accumulation is a key stressor during transport. Ammonia increased steadily with time, with the highest concentrations in controls and the lowest in MB-supplemented groups. ES + MB was particularly effective, significantly lowering ammonia by 6 h compared to all other treatments. The treatment × time interaction confirmed that ammonia suppression became significant only after 4 h, when metabolic excretion rates increased. It should be noted here that the use of commercial kits for the measurement of water quality parameters may introduce minor measurement variability; however, consistent application across treatments ensures reliable relative comparisons and has been used in previous studies [2].

The use of field-based measurement techniques and simplified microbiological methods may introduce minor variability; however, consistent application across treatments ensures the reliability of comparative trends observed in this study. It was found total bacterial counts increased markedly in controls but were strongly suppressed in MB-treated groups. By 6 h, bacterial proliferation was up to 3–4 fold higher in controls compared to MB + ES, confirming the synergistic antimicrobial effect of these additives (Tables 1, 2). The combination of salt or ES with MB consistently yielded the greatest reduction, likely due to MB’s strong antibacterial action and salt’s osmotic stress on microbes, a phenomenon similarly reported in the case of climbing perch [29]. This is also consistent with earlier observations that MB not only inhibits microbial respiration but also acts as a mild disinfectant [17, 24, 30].

Transportation stress triggers physiological responses in fish, including the release of catecholamines and corticosteroids, leading to elevated blood glucose levels [11, 31, 32]. In our study, blood glucose was significantly elevated in controls, reflecting activation of the hypothalamic–pituitary–interrenal (HPI) axis under stress. In contrast, fish in MB + ES and MB + salt groups had the lowest glucose levels, indicating mitigation of transport stress. The treatment × time interaction further confirmed that glucose differences were minimal at loading but widened progressively with transport duration (Table 3). As a quick and reliable measure of physiological stress, measurement of blood glucose level is widely recognized; however, the inclusion of additional stress indicators such as cortisol or hematological parameters would provide a more comprehensive assessment and should be considered in future studies.

Both on-farm and field trials demonstrated the superiority of additive treatments over controls, but field effects were somewhat weaker, particularly for pH regulation. This likely reflects uncontrolled environmental factors during field transport (temperature shifts and water quality variation), which can override additive benefits. Nevertheless, the consistent improvements in DO, ammonia suppression, and bacterial control across both systems underscore the robustness of MB + ES as a practical solution.

Combined treatments showed greater reductions in bacterial counts compared with single additives, suggesting a potential additive or synergistic effect. Methylene blue is widely used in aquaculture as a disinfectant at low concentrations; however, its potential residues and food safety implications should be considered. The concentration used in this study (1.0 ppm) is within commonly accepted safe limits, although further evaluation of residue dynamics is warranted. From a practical perspective, the use of salt and commercially available saline is cost-effective and easily accessible for small-scale farmers. Methylene blue, when used at low concentrations, adds minimal additional cost, making the combined treatment a feasible option for improving live fish transport.

A limitation of the present study is the lack of true biological replication at the tank level, which may limit the generalization of treatment effects. Future studies incorporating multiple independent replicates are recommended to further validate these findings. Additionally, the limited number of tanks used in the field trial reflects practical constraints of commercial transport and may affect statistical robustness. Further studies of larger sample sizes are recommended.

## 5. Conclusions

This study demonstrated that live transportation of *Pangasius hypophthalmus* without additives leads to rapid deterioration of water quality, marked by declining dissolved oxygen, acidification, elevated ammonia, and bacterial proliferation, alongside physiological stress reflected in rising blood glucose levels. Single additive treatments (salt, ES, or methylene blue alone) provided partial improvements, but their effectiveness diminished with increasing transport duration. In contrast, combined treatments consistently showed superior performance, particularly in stabilizing water quality and reducing stress responses. Among all tested combinations, 2.0 ppt ES with 1.0 ppm methylene blue emerged as the most effective, yielding significantly lower bacterial counts and ammonia accumulation, higher dissolved oxygen, and reduced glucose elevation compared with controls and other treatments. The consistency of these results under both on-farm and field conditions highlights the robustness and practical applicability of this additive strategy. These findings suggest that the combined use of ES and methylene blue has the potential to improve water quality, reduce bacterial regrowth, and mitigate stress during live transportation of Pangasius catfish.

## Data Availability

The data presented in this study are available from the corresponding author upon reasonable request.

## List of abbreviations

ANOVA, analysis of variance; AWEEC, Animal Welfare and Experimentation Ethics Committee; CFU, colony-forming unit; DO, dissolved oxygen; ES, Electromin Saline; HSD, honest significant difference; MB, methylene blue; ppm, parts per million; ppt, parts per thousand; SE, standard error; SPSS, Statistical Package for the Social Sciences; h, hours; min, minutes; °C, degrees Celsius; mg/l, milligrams per liter; mmol/l, millimoles per liter; ml, milliliter; µl, microliter; L, liter(s); kg, kilogram; CFU/ml, colony-forming units per milliliter.
